# Availability and Quality of Grief and Bereavement Care in Pediatric Intensive Care Units Around the World, Opportunities for Improvement

**DOI:** 10.3389/fped.2021.742916

**Published:** 2021-11-15

**Authors:** Michelle Grunauer, Caley Mikesell, Gabriela Bustamante, Danielle Aronowitz, Kevin Zambrano, Andrea P. Icaza-Freire, Antonio W. D. Gavilanes, Rafael Barrera, the PICU-MIC Research Group, Jorge López González

**Affiliations:** ^1^School of Medicine, Universidad San Francisco de Quito, Quito, Ecuador; ^2^Pediatric Intensive Care Unit, Hospital de los Valles, Quito, Ecuador; ^3^Department of Surgery, Long Island Jewish Medical Center, Northwell/Hofstra University School of Medicine, New Hyde Park, NY, United States; ^4^Department of Pediatrics, University Medical Center Maastricht, Maastricht, Netherlands

**Keywords:** grief, bereavement, pediatric palliative care, pediatric critical care, end of life

## Abstract

Pediatric Intensive Care Units (PICUs) provide multidisciplinary care to critically ill children and their families. Grief is present throughout the trajectory of illness and can peak around the time of death or non-death losses. The objective of this study was to assess how PICUs around the world implement grief and bereavement care (GBC) as part of an integrated model of care. This is a multicenter cross-sectional, prospective survey study. Questionnaires with multiple-choice and open-ended questions focusing on unit infrastructure, personnel, policies, limited patient data, and practices related to GBC for families and health care professionals (HCPs) were completed by on-site researchers, who were HCPs on the direct care of patients. PICU fulfillment of GBC goals was evaluated using a custom scoring based on indicators developed by the Initiative for Pediatric Palliative Care (IPPC). We compared average total and individual items fulfillment scores according to the respective country's World Bank income. Patient characteristics and details of unit infrastructure were also evaluated as potential predictors of total GBC fulfillment scores. Statistical analysis included multilevel generalized linear models (GLM) with a Gaussian distribution adjusted by child age/gender and clustering by center, using high income countries (HICs) as the comparative reference. Additionally, we applied principals of content analysis to analyze and summarize open-ended answers to contextualize qualitative data. The study included 34 PICUs from 18 countries: high-income countries (HICs): 32.4%, upper middle-income countries (UMICs): 44.1%, low middle-income and low-income countries (LMI/LICs): 23.5%. All groups reported some compliance with GBC goals; no group reported perfect fulfillment. We found statistically significant differences in GBC fulfillment scores between HICs and UMICs (specifically, HCP grief support), and between HICs and LMICs (specifically, family grief support and HCP grief support). PICUs world-wide provide some GBC, independent of income, but barriers include lack of financial support, time, and training, overall unit culture, presence of a palliative care consultation service, and varying cultural perceptions of child death. Disparities in GBC for families and HCPs exist and were related to the native countries' income level. Identifying barriers to support families and HCPs, can lead to opportunities of improving GBC in PICUs world-wide.

## Introduction

Patients, families, and healthcare providers (HCPs) experience grief and bereavement in response to loss of life or changes in quality of life (1), both of which frequently occur in pediatric intensive care units (PICUs). Evidence suggests that the traumatic experience of life-threatening illness in a child, subsequent new or worsening disability, and childhood death are all associated with increased risk of developing grief-related disorders among the bereaved (2). Both patient families and HCPs are at risk for such adverse sequelae and can experience grief differently from one another.

Numerous studies underscore the importance of providing grief and bereavement care (GBC) for the patient-family unit and HCPs (2–7), however this type of care is often inaccessible and of variable quality throughout the world (8). Inadequate GBC places families at risk of developing psychological morbidities, familial disruption, and economic hardship. Similarly, without accessible GBC, HCPs are at risk of burnout, impaired judgment, and depression (2, 3). Furthermore, data on the availability and quality of GBC in PICUs around the world is limited (9). Efstathiou et al. conducted a systematic review of bereavement support in adult ICUs in five western high-income countries (HICs) and found that this type of care was unstandardized, irregularly available, and overall insufficient to meet projected needs (8). Similarly, in their review of the need, accessibility, and quality of pediatric palliative care (PC) in low- and middle-income countries (LMICs), Sasaki et al. reported inverse relationships between country income and both GBC and PC availability (10).

Although evidence indicates that GBC is inaccessible and insufficient in most critical care units across the world (8, 9), at least one study found that units with access to in-hospital PC consultants were eight times more likely to provide GBC than those lacking these services (11). Similarly, evidence suggests that training HCP staff in PC principles as part of an integrated model of care can improve GBC accessibility (2, 3), overall quality of care (12), family satisfaction with care (13), and HCP well-being (3), perhaps even more than a PC consultation service (12, 13). In such an integrated model of care, HCP staff are trained in GBC and can identify and respond to grieving needs independent of external PC consultants (12, 14). Though less commonly used than external consultation models (12, 15, 16), the integrated model of care is increasingly described as a standard of care for seriously ill children (3, 17) and may be particularly effective in under-resourced environments (12).

To address the growing need for a standardized integrated model of pediatric PC, the Initiative for Pediatric Palliative Care (IPPC) developed a novel PC curriculum that describes six essential “domains” that inform the care of vulnerable children, their families, and their HCPs (18). The six domains are: (1) holistic care of the child; (2) support of the family unit; (3) involvement of child and family in communication, decision making, and care planning; (4) relief of pain and other symptoms; (5) continuity of care; and (6) grief and bereavement support. The sixth domain is further sub-categorized into 6A and 6B. Domain 6A consists of five actions that can be used to specifically support the child's family, including: (1) assessing the needs of the family, (2) supporting grief and bereavement-related rituals, (3) providing supportive resources, (4) employing grief and bereavement-specific support professionals and (5) instituting policies and guidelines to support the family needs. Domain 6B consists of three actions that can be used to specifically support the child's healthcare team, including: (1) establish and disseminate processes for grief and bereavement support for HCPs, (2) provide resources to address grief and bereavement needs for HCPs, and (3) have mechanisms in place to obtain feedback from grieving HCPs (13).

The objective of this multicenter cross-sectional study was to assess how PICUs around the world implement GBC as part of an integrated model of care relative to the IPPC curriculum (with a focus on domain 6). The secondary objective was to assess whether unit characteristics (physical environment, technology, and human resources), country World Bank (WB) income level (19), or patient characteristics (race, first language, age, sex, and presence of comorbidities) are associated with differences in GBC provision. Finally, this study used mixed-methods analysis to develop richer descriptions of how individual units provide GBC. Our team hypothesized that all units would at least partially comply with IPPC recommendations for GBC, independent of country income, unit characteristics, and patient characteristics.

## Materials and Methods

The international Pediatric Intensive Care Unit Model of Integrated Care (PICU-MIC) study is a multicenter cross-sectional study inclusive of 34 participating PICUs/NICUs in 18 countries. Participating centers were identified through medical societies, research networks including the Pediatric Acute Lung Injury and Sepsis Investigators Network, publication database searches, and team contacts. Each individual institution appointed a representative researcher who reviewed the study protocol and obtained local Institutional Review Board (IRB) approval. Participants were medical doctors and nurses from PICUs; HCPs not employed in PICUs were excluded. Two questionnaires with multiple choice and open-ended questions were distributed to the HCPs who were in charge of the care of each hospitalized child by the designated representative. They were distributed both in Spanish and in English. The first survey inquired about the systematic infrastructure of each unit. The second questionnaire gathered information about patient characteristics and model of care (MOC) in relation to IPPC guidelines as it applied to the care of patients who had been admitted at the time of survey distribution. Each center was requested to complete 10–25 copies of the model of care questionnaire; if centers included additional patients, we did not exclude them. A total of 498 pediatric patients were included across all centers. For each study site, 2-weeks were predefined to complete the questionnaires. Participants had a 100% response survey completion rate. We prompted survey respondents to complete the survey on REDCap which is an encrypted, password-protected online platform that allows the user to create, share, analyze and store data coming from questionnaires. Those participants who were not able to use this platform in the absence of reliable internet connection, were able to fill the de-identified questionaries via email.

The Universidad San Francisco de Quito Ethics Committee for Research of Human Beings/IRB approved this research (2016-091IN). This study was approved by Ethics Committees at all sites and its clinical trial registry number is ISRCTN12556149 (DOI 10.1186/ISRCTN12556149).

To evaluate adherence to domain 6 of the IPPC curriculum, we constructed a partial score for each item listed as subcategories under IPPC domains 6A and 6B. For each recommendation item within each subcategory, we assigned a numeric value to each answer: “yes” = 1, “sometimes” = 0.5, and “no” = 0. To create a partial score within each domain goal, we summed scores for all items and converted them to a percent such that the range of potential scores was 0–100. Lastly, a total index was created by calculating the average of the percent scores of each domain (potential final scores 1–100) (20). The arithmetic mean and standard deviation (SD) were used to summarize these scores.

To assess whether financial stability affects the availability and/or quality of GBC between institutions, we grouped institutions by income level according to the WB definitions for LMICs, UMICs, and HICs. We then compared the average scores for adherence to IPPC domain 6 among institutions at each income level. Statistical analysis included multilevel generalized linear models (GLM) with a Gaussian distribution adjusted by child age/gender and clustering by center. WB income group was modeled categorically (also using the HIC group as the reference group) and ordinally to assess the presence of a linear trend across income groups. Further, we explored whether patient or center characteristics are associated with total IPPC-adherence scores using univariate and multivariable multilevel GLM using the center as a clustering variable. The adjusted model included age, gender, race, comorbidities, and shift length. All statistical analysis was conducted using Stata v14.1. For patient characteristics, we considered age, gender, race, length of stay (LOS), diagnosis, and presence of comorbidities. For center characteristics, we considered the number of ventilators and resuscitation equipment, percent of daily bed use, beds per critical care provider (doctor or nurse), and provider shift lengths.

To better understand questionnaire answers, we also provided participants with the opportunity to provide detailed responses regarding items about GBC policies, rituals, and personnel. While the aggregate data from these open-ended questions was not detailed or extensive enough to perform an independent qualitative study, we applied concepts of content analysis to contextualize quantitative data. The results of this analysis are not generalizable but provide richness to our study results and may help orient further research and clinical considerations. We also applied concepts of content analysis and grounded theory as part of a mixed-methods methodology to analyze the participants' open-ended responses. After extracting and categorizing answers by question, answers were stratified by WB income level. Next, we assigned responses to categories, eliminated duplicates, and summarized responses when possible. Categories included: (1) support of, engagement with, and attitudes about patient-family GBC rituals, (2) individuals with experience in grief and loss available to provide GBC support, and (3) policies and guidelines established to ensure grief and loss support is provided to patients and families by country income level. Then, we compared original participant responses to ensure each answer was represented. Finally, we compared differences in the participants' answers according to WB country income level in order to connect data provided in open-ended responses to findings from our statistical analysis and literature review. We have included tables and a summary of results regarding centers' support of rituals, GBC facilitators, and GBC policies/guidelines by country income level group (Supplementary Material).

## Results

The PICU-MIC collaboration included 34 participating PICUs from 18 countries across Asia (15), Latin America (7), North America (5), Europe (5), and Africa (2). HIC units made up 32.4% of the sample, UMICs made up 44.1%, and lower middle-income/lower-income countries LMIC/LICs made up 23.5%.

Across all centers, fulfillment of the IPPC recommendations in offering family-specific grief and bereavement support (goal 6A) reached an average score of 37.5% (SD: 28.1) (Table 1). Scores increased with respect to income group, and ranged from 22.1% among the LIC/LMICs to the highest average score of 48.0% among HICs (*p*-value for trend: 0.02). This trend was not observed for each individual indicator of goal 6A. We found that the availability of appropriate services to support grief and bereavement of the family was higher among units in HICs (61.4%) compared to LICs (24.5%, *p*-value for trend: 0.004). Similarly, policies and guidelines for grief support were more often reported by units in HICs vs. LIC/LMICs (31.9 vs. 3.0%, *p*-value for trend: 0.001).

**Table 1:** Grief and bereavement support goals (6A and 6B) and indicators according to World Bank income level classification.

**Goals and indicators**	**Low and lower middle income**	**World bank income level**		
		**Upper middle income**	**High income**	**All centers**
	**Mean (sd)^a^** ***p-*trend^b^**	**Mean (sd)^a^**	**Mean (sd)^a^**	**Mean (sd)^a^**
**6A—Family**				
**Overall**	25.1 (21.6) **0.02**	34.9 (23.8)	48.0 (32.2)	37.5 (28.1)
Family grief needs	49.0 (47.1) 0.89	46.0 (44.9)	49.4 (48.2)	47.8 (46.0)
Support of rituals	36.5 (43.7) 0.75	69.8 (41.6)	50.0 (49.1)	56.3 (46.6)
Grief support resources	24.5 (37.9) **0.004**	33.0 (46.2)	61.4 (45.4)	41.1 (46.8)
Grief professional support	12.5 (32.1) 0.09	17.0 (35.6)	43.9 (46.7)	25.3 (41.4)
Policies for grief support	3.0 (15.6) **0.001**	4.8 (21.0)	31.9 (44.6)	13.8 (33.2)
**6B—Health care providers**				
**Overall**	22.3 (24.0) **0.001**	32.8 (29.1)	64.1 (35.8)	42.1 (35.3)
Processes for HCP grief support	19.5 (31.7) **0.02**	18.7 (35.7)	64.0 (45.5)	34.3 (44.1)
Resources for HCP grief support	17.0 (28.6) **0.002**	21.1 (40.4)	69.3 (43.8)	36.8 (46.0)
Feedback from grieving HCP	28.5 (41.6) 0.08	41.6 (42.0)	54.7 (47.0)	43.5 (44.7)

a *Mean and standard deviation (sd), Scores range from 0 to 100%-points*.

b *p-trend: p-value for linear trend estimated using GLMs adjusted for child's age and gender, and using the center as a clustering variable. HCP, health-care provider. The bold values are statistically significant with a p-value of < 0.05*.

Centers achieved an average fulfillment score of 42.1% (SD: 35.3) for the IPPC recommendation to offer grief and bereavement support for HCPs (goal 6B, Table 1). Similarly, overall scores for goal 6B increased from 22.3% in LIC/LMICs to 64.1% in HICs (*p*-value for trend: 0.001). However, unlike goal 6A, we found evidence of an increasing trend in scores for each individual indicator of goal 6B as detailed in Table 1. Institutions located in HICs more frequently reported the existence of processes, resources and feedback mechanisms to support grieving HCPs (*p*-value for trend: 0.02, 0.002, and 0.08, respectively).

Associations between overall adherence scores for goals 6A and 6B and both sociodemographic characteristics of the patients and structural characteristics of the centers were identified and summarized in Table 2. For goal 6A (grief and bereavement support of the family), we found that units reported higher scores for children with PICU stays longer than a month compared to children with shorter stays (adjusted *p*-value: 0.03). Similarly, units reported higher levels of grief and bereavement support for families of children with multiple vs. single morbidities (adjusted *p*-value: 0.002). In terms of center characteristics, the only factor associated with fulfillment of goal 6A was the length of shifts but not the availability of equipment or specialized personnel. After adjusting for patient demographics and other center characteristics, scores consistently decreased as shift lengths increased (adjusted *p*-value for trend: 0.03).

**Table 2:** Associations between patient and center characteristic, and overall scores for domains 6A and 6B of the Initiative for Pediatric Palliative Care's recommendations.

**Characteristics of the patient**	**Family GBC**			**HCP GBC**		
	**Mean (sd^a^)**	***P-*value**	**Adj. *P-*value**	**Mean (sd)**	***P-*value**	**Adj. *P-*value**
**Age**						
Newborn (0–1 m) *N* = 33 (7.2%)	34.4 (28.8)	0.62	0.93	36.8 (39.1)	0.69	0.96
Infant (>1–12 m) *N* = 113 (24.5%)	35.9 (26.9)	0.23	0.54	38.7 (32.8)	**0.04**	**0.02**
Preschool (>1–5 y) *N* = 142 (30.8%)	39.6 (27.6)	Ref.^a^	Ref.	41.1 (37.6)	Ref.	Ref.
School (>5–11 y) (*N* = 92) (20%)	34.5 (26.3)	0.84	0.48	32.5 (35.7)	0.40	0.30
Teen (>11–18 y) *N* = 81 (17.6%)	37.1 (26.8)	0.91	0.88	40.1 (34.5)	0.40	0.56
**Gender**						
M, *N* = 262 (56.8%)	36.3 (27.8)	Ref.	Ref.	35.1 (34.3)	Ref.	Ref.
F, *N* = 199 (43.2%)	37.6 (26.1)	0.65	0.90	42.4 (36.9)	0.56	0.41
**Race**						
White, *N* = 165 (35.9%)	44.2 (29.3)	Ref	Ref.	50.4 (39.8)	Ref.	Ref.
Asian, *N* = 82 (17.8%)	30.7 (20.9)	0.64	0.35	20.4 (23.0)	0.55	0.87
Black, *N* = 54 (11.7%)	25.4 (27.3)	0.71	0.82	41.0 (36.4)	**0.01**	**0.001**
Indian, *N* = 31 (6.7%)	37.4 (9.99)	0.77	0.42	18.3 (18.9)	0.41	0.3
Mixed-race, *N* = 57 (12.4%)	37.5 (34.1)	0.66	0.61	33.6 (29.3)	0.31	0.48
Middle eastern, *N* = 67 (14.6%)	37.9 (23.2)	0.60	0.92	45.8 (36.3)	0.86	0.76
Other, *N* = 4 (0.9%)	10.0 (20.0)	0.20	0.26	50.0 (19.2)	0.71	0.56
**Days in PICU**						
<30 days, *N* = 390 (84.6%)	36.1 (27.11)	Ref.	Ref.	38.3 (36.2)	Ref.	Ref.
>30 days, *N* = 71 (15.4%)	41.5 (26.8)	**0.05**	**0.03**	38.3 (32.3)	0.69	0.21
**Comorbidities**						
Single MDC^b^, *N* = 126 (27.6%)	36.2 (26.9)	Ref.	Ref	36.7 (35.8)	Ref.	Ref.
Multiple, *N* = 331 (72.4%)	37.1 (27.2)	**0.01**	**0.002**	38.8 (35.6)	**0.01**	**0.002**
**Characteristics of the center**						
**Number of ventilators**						
<1 per bed, *N* = 201 (43.6%)	34.7 (22.9)	Ref.	Ref.	32.6 (33.6)	Ref.	Ref
>1 per bed, *N* = 260 (56.4%)	38.8 (30.3)	0.89)	0.52	43.4 (36.7)	0.91	0.12
**Resuscitation equipment**						
<0.5 per bed, *N* = 323 (70.1%)	37.4 (26.5)	Ref.	Ref.	38.1 (38.4)	Ref.	Ref.
>0.5 per bed, *N* = 138 (29.9%)	35.4 (28.4)	0.95	0.23	38.8 (27.2)	0.99	0.98
**Percent daily bed use**						
<80%, *N* = 139 (30.2%)	39.7 (28.5)	Ref.	Ref.	45.6 (43.8)	Ref.	Ref.
>80%, *N* = 322 (69.88%)	35.3 (26.2)	0.77	0.59	34.2 (29.5)	0.50	0.14
**Beds/critical care doctor**						
<2 beds per doctor, *N* = 282 (65.7%)	36.1 (27.2)	Ref.	Ref.	38.3 (37.2)	Ref.	Ref.
>2 beds per doctor, *N* = 147 (34.3%)	38.0 (21.7)	0.80	0.80	38.9 (34.6)	0.64	0.75
**Beds/nurse**						
<2 beds per nurse, *N* = 208 (50.7%)	41.1 (30.3)	Ref.	Ref.	39.5 (39.1)	Ref.	Ref.
>2 beds per nurse, *N* = 202 (49.3%)	30.6 (23.1)	0.15	.83	35.9 (32.6)	0.55	0.64
**Shift length**						
<8 h, *N* = 102 (22.1%)	59.2 (24.4)	Ref.	Ref.	50.0 (41.4)	Ref.	Ref.
8–12 h, *N* = 241 (52.3%)	34.2 (28.3)	**0.01**	**0.001**	43.0 (33.3)	0.73	0.99
13–18 h, *N* = 42 (9.1%)	27.1 (26.2)	**0.02**	**0.009**	50.8 (40.8)	0.95	0.52
19–24 h, N=20 (4.2%)	12.0 (12.8)	**0.001**	**0.001**	17.5 (18.3)	0.17	0.30
*Multiple, N* = 56 (12.2%)	29.0 (8.61)	**0.01**	**0.008**	11.8 (13.4)	**0.04**	**0.02**
*p-*value *for trend*		**0.01**	**0.03**		**0.02**	**0.02**

*^‡^ p-values were estimated using univariate and multivariable multilevel GLMs using center as a clustering variable. The adjusted model considers all factors in the table*.

a *sd, standard deviation; Ref, reference category*.

b *MDC, Major diagnostic category. The bold values are statistically significant with a p-value of < 0.05. ‡P-value headers*.

For goal 6B (GB support of HCPs), we found an association between overall scores and patients' age and race without a clear pattern (Table 2). Units reported slightly higher levels of GBC for HCPs who cared for patients with multiple comorbidities compared to patients with a single morbidity (adjusted *p*-value: 0.002). In general, we did not find that center personnel or infrastructural characteristics were associated with the fulfillment of goal 6B. However, units with longer shift lengths had lower scores in goal 6B than units with shorter shift lengths (adjusted *p*-value for trend: 0.02).

Regarding detailed responses about policies, rituals, and personnel for grief and bereavement support, some participants in HICs mentioned that families are allowed as much time as they consider needed for their morning process and rituals. Others mentioned that all cultural/religious rituals and beliefs can take place as long as it is not life-threatening to the patient. Personnel from UMICs centers had similar responses. Answers pertaining which individuals with experience in grief and loss care were available to provide support varied greatly among centers from HICs, UMICs or LMICs/LICs. While responses coming from HICs included a wide variety of available personnel like psychologists, intensivists, nurses, rehabilitation services, pain management teams, social workers, interpreters and others, responses from LMICs/LICs included only a handful of those experts. Lastly, participants from HICs and UMICs mentioned the existence and use of specific guidelines dedicated to ensuring grief and loss support in their centers, despite considering them lacking in some instances. Contrastingly, LMICs/LICs did not specify any guidelines used.

## Discussion

This international multicenter study revealed a statistically significant inverse relationship between country income level and the availability and quality of GBC to PICU patients, families, and HCPs. These findings echo what is already known from the literature (10) and reveal the precise aspects of GBC which vary between countries of different income levels in our sample. Similarly, a survey made in 2010 in 58 countries found that bereavement care for pediatric oncologic patients was available only in 28.3% of their sample with a statistically significant difference by income level in availability and existence of laws or institutional policies regarding GBC (21).

Regardless of income level, about half of all centers reported that they asked families about their needs for GBC both during the child's hospitalization and after their death. However, access to supportive resources for the family-patient unit, including specially trained staff and holistic care related to death and disease, varied greatly between centers (Table 1). Other studies have also found that most HCPs consider this type of service as necessary for pediatric palliative care practice, including those working in LMICs/LICs, despite having a different availability of resources to do so (21). In our sample, UMICs reported the highest amount of GBC rituals available to families, although mixed-methods analysis showed that HICs described providing more types of rituals, having more diverse on-site professional support, and generally more active participation in rituals. Participants generally reported support for diverse end of life rituals as long as these did not risk the well-being of the patient or others. Participants sometimes saw the facilitation of rituals as the responsibility of other specialists (e.g., chaplain, psychologist, religious leaders), though others described the accommodation and regulation of rituals as an important facet of intensive care. Some respondents specified that rituals were restricted to cases of imminent death, prior to death, and upon request.

With respect to timing of GBC, standard of care guidelines dictate that PC should be available from the moment of diagnosis (22), and not reserved for instances of imminent death as reported by some centers. Furthermore, evidence shows that rituals may improve family and HCP capacity to cope with the devastating situation, accept unanticipated losses, experience positive feelings following grief, and restore feelings of control (23, 24). Future studies are needed to further determine the best mechanisms of implementing and standardizing GBC such that hospital resources are allocated efficiently to optimize patient-family outcomes.

This study also identified differences in the availability of professionals to provide GBC by country level income. While the differences did not reach statistical significance, HICs (43.9) reported the greatest availability of GBC professionals, followed by UMICs (17.0), and then LMICs/LICs (12.5). This trend was reflected in the mixed-methods analysis (Table 1). Overall, HICs described having more availability of multidisciplinary professionals working in their centers in comparison to UMICs, which, in turn, reported more multidisciplinary professionals available than LMICs/LICs.

Additionally, we observed differences in the use of established policies and guidelines for GBC according to country income level. This finding was also reflected in the mixed methods analysis. HICs were most likely to report using established GBC policies/guidelines and reported a greater variety of standardized policies than units of other income levels. Notably, although not explicitly asked, professionals in UMICs reported disagreements and worries about the suitability of official guidelines in their units (e.g., lack of universal applicability, lack of standardization, a complete absence of guidelines, or not knowing if there were guidelines). UMICs also reported the use of more unit-specific and non-standardized guidelines than HICs. Some units in both HICs and UMICs reported substitutes (e.g., experience, routines, “tacit agreements”) for the use of standardized guidelines. Other participants saw grief and loss support as the responsibility of other departments. LMICs/LICs did not specify the policies/guidelines used.

Evidence shows that standardizing many facets of critical care may improve outcomes, reduce care costs, and minimize length of stay, but practices to ensure standardization of care are not widely implemented (25). The establishment of GBC guidelines that are acceptable to professionals especially for centers in UMICs, LMICs, and LICs may represent a low-cost method to improving quality of care, patient-family outcomes, and satisfaction with care. Furthermore, evidence suggests that, particularly in LMICs, local government and community organizations can improve the availability and quality of grief and bereavement support in the healthcare system by supporting the implementation of such guidelines (10).

Our study also found differences in GBC for HCPs by country income level. Overall, HICs reported more diverse opportunities supporting healthcare professionals to express their GBC needs, more formalized services, and more regular support opportunities than units in other income groups. Important variations in support opportunities included the frequency of opportunities (e.g., regularity, formality, and prioritization of care opportunities), specialization of facilitators (e.g., psychologists, trained peers, informal support between colleagues), cost of services (e.g., free, independently paid, or unspecified), nature of opportunities (e.g., preventative vs. reactionary), and accessibility (e.g., regularly or sporadically available, unregulated informal support online, 24/7 hotlines). Participants similarly described differences in the resources dedicated to staff (e.g., reserved time, space, professionals) and diversity of services (e.g., only one type of support, or a combination of psychological, religious, spiritual, social work, general health, social, other support). Finally, participant responses reflected a diversity of attitudes regarding how HCPs are perceived by others with regard to their GBC needs (e.g., second victims, professionals) and who is responsible for providing GBC (e.g., individual, team/unit, institution). Normalizing and formalizing GBC for PICU professionals is important because unaddressed grief among HCPs may contribute to maladaptive coping, unhealthy work environments, burnout, and other psychosocial issues (26), as well as ultimately affect the patient-family unit's quality of care (2, 3).

While the present study was completed prior to the COVID-19 pandemic, evidence suggests a dramatically growing need for GBC for patients, families, and HCPs (27) affected by COVID-19. The disparities and insufficiencies of GBC in PICUs around the world highlighted in our research will likely worsen as a result of this international crisis. Our findings highlight the need to develop interventions to improve the GBC for patients, families, and PICU professionals, irrespective of country income.

Our study has strong points. We analyzed various possible variables determining grief and bereavement care. These included unit characteristics, human resources, patient characteristics and country World Bank Income classification. In addition, we included data from centers located in areas which are not frequently considered in scientific research, either due to geographic or resource limitations or due to language barriers. However, our study has limitations. Responses coming exclusively from centers are not a reliable representative for IPPC curriculum adherence in the whole country. Moreover, we did not request the involved institutions to declare whether they were from urban, suburban or rural areas. Neither did we ask to specify if the centers had public, private or public-private funding. Furthermore, opinions on availability and provision of grief and bereavement care may vary depending on the seniority of the medical professional answering the questionnaires and we did not include this variable in our survey. Finally, determining GBC fulfillment exclusively via assessment of the IPPC curriculum may not be fully representative of how this service is practiced and offered in the countries evaluated.

## Conclusion

Independent of ultimate patient outcomes, the experience of PICU hospitalization is associated with diverse psychosocial and physical sequelae amongst pediatric patients and their families (28). HCPs are also at risk for burnout, psychiatric illness, etc. The often-undertreated grief and bereavement needs of patients, families, and HCPs are intertwined with the development of these sequelae and thus merit standardized attention within in the PICU. The present study highlighted disparities in GBC provision for both the patient-family unit and HCPs in PICUs across the globe. Accessibility and quality of GBC were inversely related to country income level. Furthermore, our mixed-methods analysis identified specific care techniques used by different PICUs around the world as well as future areas of research. Thus, we provide evidence related to ways in which care practices may vary by country income group as well as points of consideration for further research.

## Data Availability Statement

The original contributions presented in the study are included in the article/Supplementary Material, further inquiries can be directed to the corresponding author/s.

## Ethics Statement

The studies involving human participants were reviewed and approved by Universidad San Francisco de Quito Ethics Committee for Research of Human Beings/IRB. Written informed consent for participation was not required for this study in accordance with the national legislation and the institutional requirements.

## PICU-MIC Investigators

1. Jorge López González, MD; Jesús López-Herce, MD—Hospital General Universitario Gregorio Marañón-UCIP; Facultad de Medicina Universidad Complutense de Madrid, Madrid, Spain.
2. Emanuele Rossetti, MD; Chiusolo Fabrizio, MD—Ospedale Pediatrico Bambino Gesù-PICU, Roma, Italy.
3. Oliver Karam, MD, PhD, Marie Saint-Faust, MD—Geneva University Hospitals-HUG PICU, Geneva, Switzerland.
4. Paolo Biban, MD; Silvia Carlassara, MD—Azienda Ospedaliera Universitaria Integrata Verona- PICU, Verona, Italy.
5. Bettina von Dessauer, MD; Nadia Ordenes, MD—Hospital de Niños Roberto del Río Unidad de Paciente Crítico Pediátrico, Santiago, Chile.
6. Fabiola Figueroa Urízar, MD; Adriana Wegner A, MD—Hospital Sótero Del Río-UCIP, Santiago, Chile.
7. Michael Canarie, MD—Yale-New Haven Children's Hospital's Pediatric Intensive Care Unit, New Haven, Connecticut, USA.
8. Kathryn Miller, MD—University Hospitals Cleveland Medical Center-PICU, Cleveland, Ohio, USA.
9. José Irazuzta, MD; Nicolas Chiriboga, MD—University of Florida COM-PICU, Jacksonville, Florida, USA.
10. Daniel Tawfik, MD, MS; Barbara Sourkes, PhD; Nancy Ewen Wang, MD; Hursuong Vongsachang—Division of Pediatric Critical Care Medicine at Stanford University Medical Center-PICU, Stanford, CA, USA.
11. Elizabeth W. Tucker, MD; Nicole Shilkofski, M.D., M.Ed.; The Johns Hopkins Children's Center PICU, Baltimore, MD, USA.
12. 王文超 Wang Wenchao, RN; Zhang Yuxia, RN, PhD; Pediatric Hospital of Fudan University-PICU—Fudan, China.
13. Lucy Lum Chai See, MD; Sister Priscilla—University Malaya Medical Center-PICU, Kuala Lumpur, Malaysia.
14. Recep Tekin, MD; Fesih Aktar, MD—Dicel University Medical Hospital-PICU; Diyarbakir, Turkey
15. Duygu Sönmez Düzkaya, CPN, PhD—Istanbul University Faculty of Medicine PICU; Istanbul, Turkey.
16. Oguz Dursun, MD; Ebru Atike Ongun, MD—Akdeniz University-Faculty of Medicine PICU; Antalya, Turkey.
17. Resul Yilmaz, MD—Gaziosmanpasa University School of Medicine Tokat- PICUTokat, Turkey.
18. Dincer Yildizdas, MD—Çukurova University, Faculty of Medicine; Balcali Hospital, PICU- Adana, Turkey.
19. Hakan Tekgüç, MD—Koru Hospital-PICU; Ankara, Turkey.
20. Vitaliy Sazonov, MD; Timur Tsoy, MD—PICU of City Children Hospital; Astana, Kazakhstan.
21. Vitaliy Sazonov, MD; Askhat Saparov, MD—PICU of National Research Center for Maternal and Child Health; Astana, Kazakhstan.
22. Vitaliy Sazonov, MD; Elizaveta Kalmbakh, MD—PICU of Karaganda Regional Children's Hospital, Karaganda, Kazakhstan.
23. Michelle Grunauer, MD, PhD (USFQ/HDLV); Ernesto Quiñones, MD (HDLV); Luis Eguiguren, MD (USFQ); Killen Briones, MD (UG, CFRBC, ICU, ICU IESS); UCIP/UCIN del Hospital de los Valles, Quito, Ecuador; Universidad San Francisco de Quito, Facultad de Medicina, Quito, Ecuador; Universidad de Guayaquil, Facultad de Ciencias Médicas, Guayaquil, Ecuador; Centro Fisiológico-Respiratorio Briones Claudett, Guayaquil; ICU Panamerican Clinic, Guayaquil; ICU IESS de Babahoyo, Ecuador.
24. Yaneth Tovilla, MD—Unidad Pediátrica de Quemados de los Servicios de Salud del Estado de Puebla, Puebla, México.
25. Sandra Tania Ventura Gómez, MD—UCIP del Hospital Regional de Alta Especialidad de Ixtapaluca, Ixtapaluca, México.
26. Silvio Fabio Torres, MD, MSc—Pediatric Intensive Care Unit, Hospital Universitario Austral; Buenos Aires, Argentina.
27. Paul Cobarrubias, MD—Amang Rodriguez Memorial Medical Center-PICU, Manila, Philippines.
28. Dmytro Dmytriiev, MD—Vinnitsa National Medical University-PICU, Vinnitsa, Ukraine.
29. Alejandro Martínez, MD; Gustavo Guzaman, MD; Rudy Sanabria, MD—Hospital del Niño Manuel Ascencio Villarroel, Cochabamba-UTIP, Cochabamba, Bolivia.
30. Ravikumar Krupanandan, MD; Bala Ramachandran, MD—PICU, Kanchi Kamakoti CHILDS Trust Hospital, Chennai, India.
31. Nirmal Choraria, MD; Jignesh Patel, MD; PICU—Nirmal Hospital, Ltd.; Surat, India.
32. Puneet A Pooni, MD; Karambir Singh Gill, MD—Dayanand Medical College & Hospital-PICU; Punjab, India.
33. John Adabie Appiah, MD; Komfo Anokye Teaching Hospital—PICU, Kumasi, Ghana.
34. Tigist Bacha Heye, MD; Rahel Argaw, MD; Asrat Demtse, MD; Israel Abebe Admasu, MD—Addis Ababa University, College of Health Sciences-PICU, Addis Ababa, Ethiopia.

## Author Contributions

MG, CM, and GB contributed to, substantial contributions to the conception or design of the work and the acquisition, analysis, or interpretation of data for the work, critical revision for important intellectual content, final approval of the version to be published, and agreement to be accountable for all aspects of the work in ensuring that questions related to the accuracy or integrity of any part of the work are appropriately investigated and resolved. DA: contributed to, critical revision for important intellectual content, final approval of the version to be published, and agreement to be accountable for all aspects of the work in ensuring that questions related to the accuracy or integrity of any part of the work are appropriately investigated and resolved. KZ contributed to, literature review, wrote the first draft, and agreement to be accountable for all aspects of the work in ensuring that questions related to the accuracy or integrity of any part of the work are appropriately investigated and resolved. AI-F, AG, and RB contributed to, critical revision for important intellectual content, and agreement to be accountable for all aspects of the work in ensuring that questions related to the accuracy or integrity of any part of the work are appropriately investigated and resolved. PICU-MIC investigators: substantial contributions to the conception or design of the work and the data acquisition. All authors contributed to the article and approved the submitted version.

## Funding

This project was funded by Universidad San Francisco de Quito, Collaboration and Medical School's Grants.

## Conflict of Interest

The authors declare that the research was conducted in the absence of any commercial or financial relationships that could be construed as a potential conflict of interest.

## Publisher's Note

All claims expressed in this article are solely those of the authors and do not necessarily represent those of their affiliated organizations, or those of the publisher, the editors and the reviewers. Any product that may be evaluated in this article, or claim that may be made by its manufacturer, is not guaranteed or endorsed by the publisher.
