# Supplementary material for: Availability and Quality of Grief and Bereavement Care in Pediatric Intensive Care Units Around the World, Opportunities for Improvement
Source: Front Pediatr. 2021 Nov 15;9:742916. doi: 10.3389/fped.2021.742916 (PMC8634722; doi:10.3389/fped.2021.742916)
Supplement: Supplementary file 1 [file Data_Sheet_1.PDF]

QUESTIONNAIRE #2

CODE ASSIGNED TO PATIENT:

SEX:

Male:

Female:

AGE:

RACE:

FIRST LANGUAGE(S):

ADMISSION DIAGNOSIS: (if more than one please list all)

DAYS IN THE PICU:

Personnel filing out the questionnaire:

Nurse Attending Resident Medical student NP PA

DOMAIN 1

- Are the needs of the child assessed in combination with him/her and his/her family on a regular basis? YES NO Sometimes N/A

Physical	YES	NO	Sometimes	N/A
Social	YES	NO	Sometimes	N/A
Emotional	YES	NO	Sometimes	N/A
Developmental	YES	NO	Sometimes	N/A
Spiritual	YES	NO	Sometimes	N/A
Educational	YES	NO	Sometimes	N/A

If necessary, include other examples:

- Is the assessment focused on the child's needs according to their developmental stage and diagnosis?

YES NO Sometimes N/A

- Based on this assessment, are appropriate interventions executed with cooperation of the child and family?

YES NO Sometimes N/A

If yes, how often:

Please provide an example:

- The development and implementation of interventions involves any of the following:

• Chaplaincy:	YES	NO	Sometimes	N/A
Physical and occupational therapy:	YES	NO	Sometimes	N/A
Psychology:	YES	NO	Sometimes	N/A
Palliative medicine team:	YES	NO	Sometimes	N/A
Bioethics personnel:	YES	NO	Sometimes	N/A
Others:	YES	NO	Sometimes	N/A

Which ones?

- Are there policies and guidelines to make sure the above indicators are carried out?

YES NO Sometimes N/A

If yes, please provide an example:

DOMAIN 2

- Do you consider the child and his/her family as a unit of care?

YES NO Sometimes N/A

- Parental concerns are addressed and acknowledged?

YES NO Sometimes N/A

How often?

- The meaning of family to the child and its members and their family dynamic is respected by the institution and healthcare providers?

YES NO Sometimes N/A

If yes, please provide an example:

- Are cultural values, beliefs, and points of view regarding quality of life from the child/'s family and the community respected?

YES NO Sometimes N/A

If yes, please provide an example:

- Is the family involved based on their personal requests and the child's condition?

YES NO Sometimes N/A

If yes, please provide an example:

- Is the impact that the child's condition has on siblings and other family members assessed routinely and strategies for coping developed?

YES NO Sometimes N/A

If yes, how often:

- Is there financial, emotional, and spiritual support available through the institution for family members?

YES NO Sometimes N/A

If yes, please provide an example:

DOMAIN 3

- Is the decision making ability of the child assessed?

  YES NO Sometimes N/A
- If yes, is this documented in the patient chart:

  YES NO Sometimes
- How often:

- Is appropriate information shared with the child regarding his/her condition and treatment strategies, based on the child's developmental stage?

  YES NO Sometimes N/A

- Are any of the following offered to the child?

The option to choose when and where procedures are performed:

YES NO Sometimes

The option to choose parental presence for support:

YES NO Sometimes

The use of sedation:

YES NO Sometimes

The choice of non pharmacologic techniques for pain and stress management:

YES NO Sometimes

- Are the perspectives and needs of the child assessed and incorporated in his/her plan of care?

  YES NO Sometimes N/A

If yes, how often:

- Are there consent forms and tools available that are appropriate for the child's age?

  YES NO Sometimes N/A

If yes, please provide an example:

- Is the family provided with information regarding the child's condition, treatment, and use of experimental protocols?

YES NO Sometimes N/A

If yes, how often:

Under request: YES NO Sometimes

- Is the PICU personnel familiar with national guidelines regarding ethical and legal issues in palliative care? YES NO

If yes, which are those:

- Are there mechanisms available to solve ethical dilemmas and to handle differences of opinion between family, child and PICU personnel regarding goals and benefits of treatment?

YES NO Sometimes N/A

If yes, which are these:

DOMINIO 4

- Are pain and other symptoms assessed?

YES NO Sometimes

If yes, how often:

Using which methods:

- Is this documented in the patient chart?

YES NO Sometimes

- Pain assessment is focused on:

Expressed pain: YES NO Sometimes

Observed pain: YES NO Sometimes

Physiological indicators: YES NO Sometimes

Family reports: YES NO Sometimes

Ability of the child to participate in activities of daily living:

YES NO Sometimes

- Is an appropriate treatment plan (including a range of pharmacological and non-pharmacological interventions), formulated, re-assessed and changed accordingly with the child and family included?

YES NO Sometimes N/A

If yes, how often:

- Are individuals with expertise in pain management involved in the child's management?

YES NO Sometimes N/A

If yes, who:

- Are there policies and guidelines to ensure that pain and other symptom management are optimally handled?

YES NO Sometimes N/A

If yes, which ones?

DOMAIN 5

- Is there a process that involves the child, family and PICU personnel in the elaboration of a written integral plan of care?

YES NO Sometimes N/A

- Is there a timely and appropriate communication regarding the integral plan of care?

YES NO Sometimes N/A

- Is there a continuous revision of the plan of care (and modifications) with the child and family? YES NO Sometimes N/A

- Does the healthcare provider involve the child, family and other personnel in the planning and decision making process? YES NO Sometimes

- Is there constant communication between the above mentioned people?

  YES NO Sometimes

- Is there the possibility of a change in healthcare provider if the child or family requests? YES NO Sometimes N/A

DOMAIN 6

- Is the family asked about their needs for grief and loss, during the child's disease and after death?

  YES NO Sometimes N/A

- Is the opportunity given to the child and family to perform rituals based on beliefs regarding death and disease?

  YES NO Sometimes N/A

If yes, please provide and example:

- Are there appropriate resources to support the child's family?

  YES NO Sometimes N/A

- Are individuals with experience in grief and loss involved?

  YES NO Sometimes N/A

If yes, who?

- Are there policies and guidelines established to ensure grief and loss support is provided?

  YES NO Sometimes N/A

If yes, please provide an example:

- Are there processes and opportunities for healthcare providers to express their feelings about grief and their support needs?

YES NO Sometimes N/A

If yes, please provide an example:

- Are there resources available for the physical, emotional and spiritual needs of healthcare providers?

YES NO Sometimes N/A

If yes, please provide an example:

- Is there feedback from healthcare providers regarding the availability and efficiency of resources at an institutional level?

YES NO Sometimes N/A
