# Supplementary material for: Availability and Quality of Grief and Bereavement Care in Pediatric Intensive Care Units Around the World, Opportunities for Improvement
Source: Front Pediatr. 2021 Nov 15;9:742916. doi: 10.3389/fped.2021.742916 (PMC8634722; doi:10.3389/fped.2021.742916)
Supplement: Supplementary file 2 [file Table_1.DOCX]

**Support of, engagement with, and attitudes about patient-family GBC rituals:**


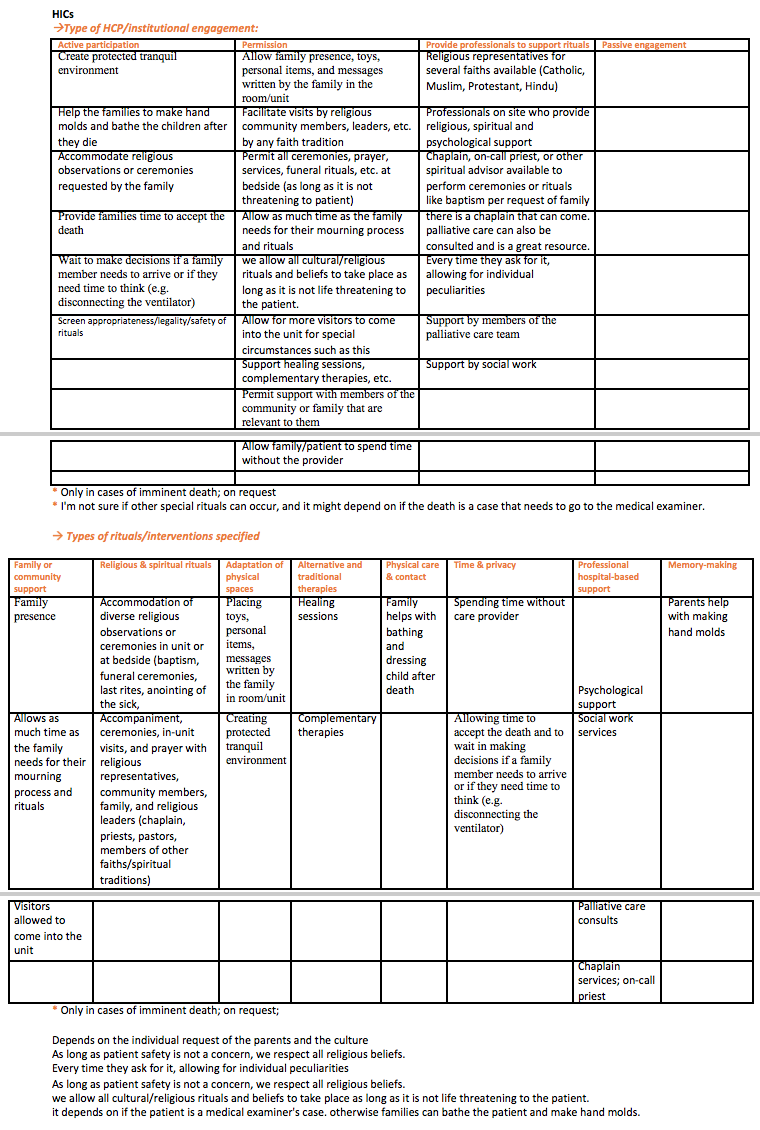


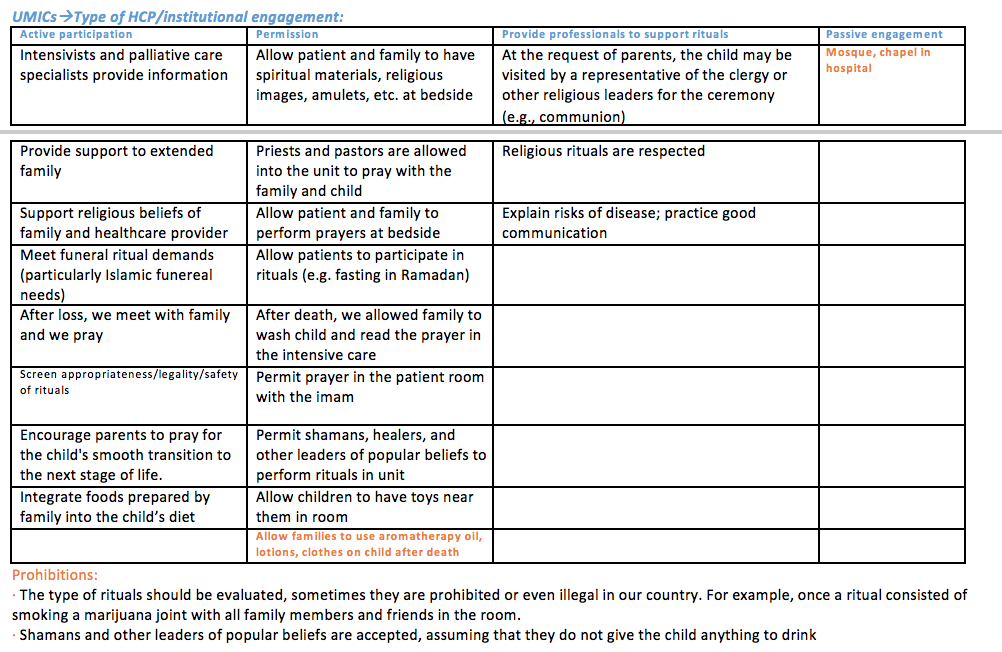


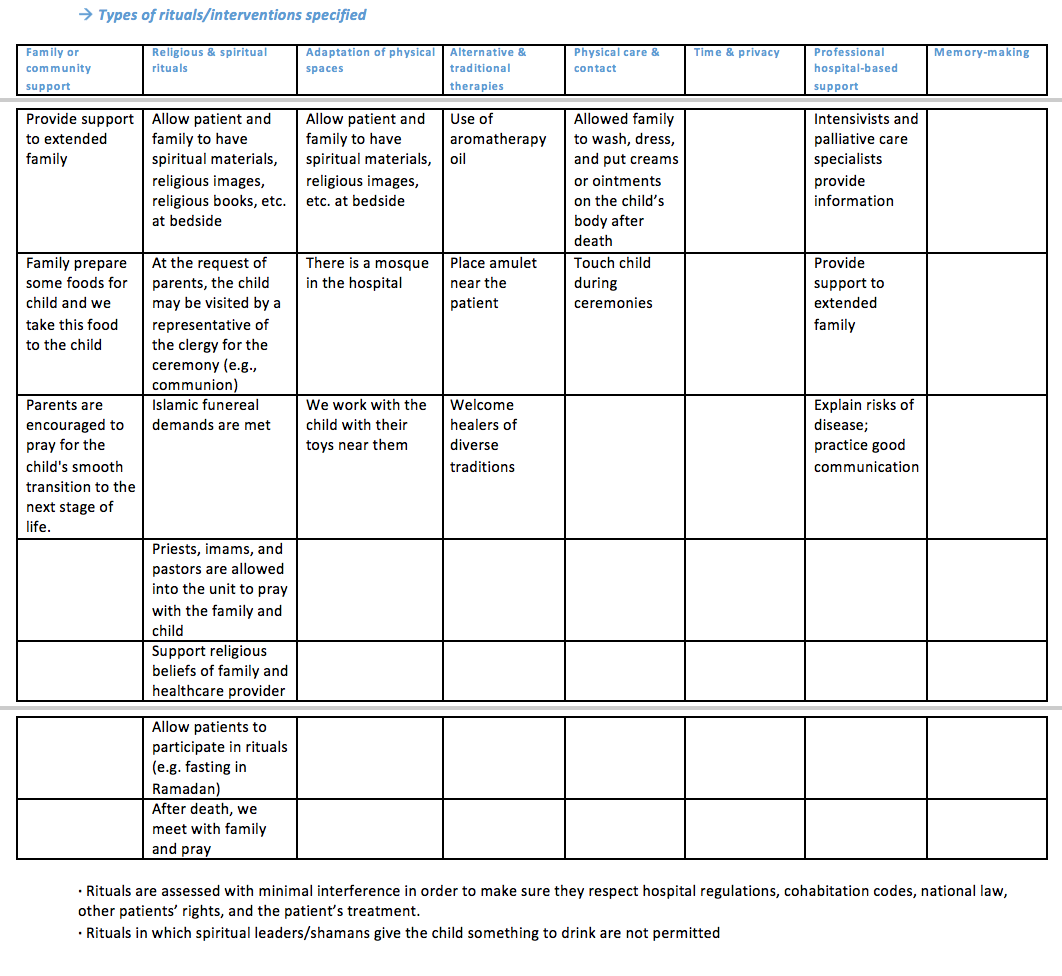


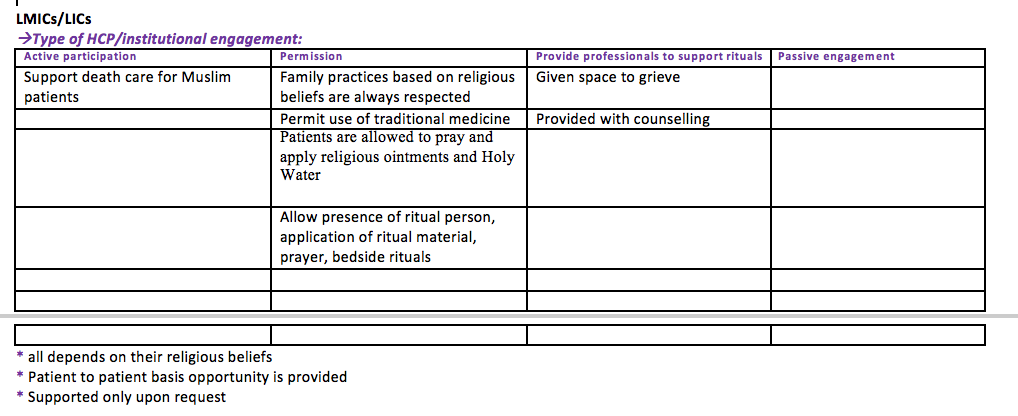


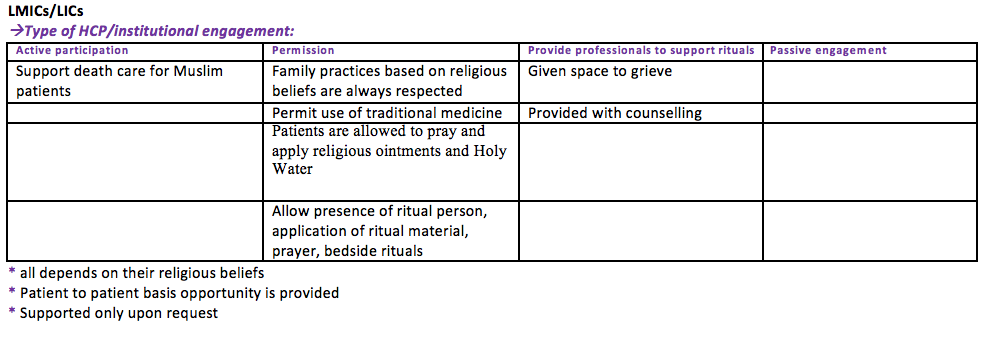


**Individuals with experience in grief and loss available to provide GBC support:**

**
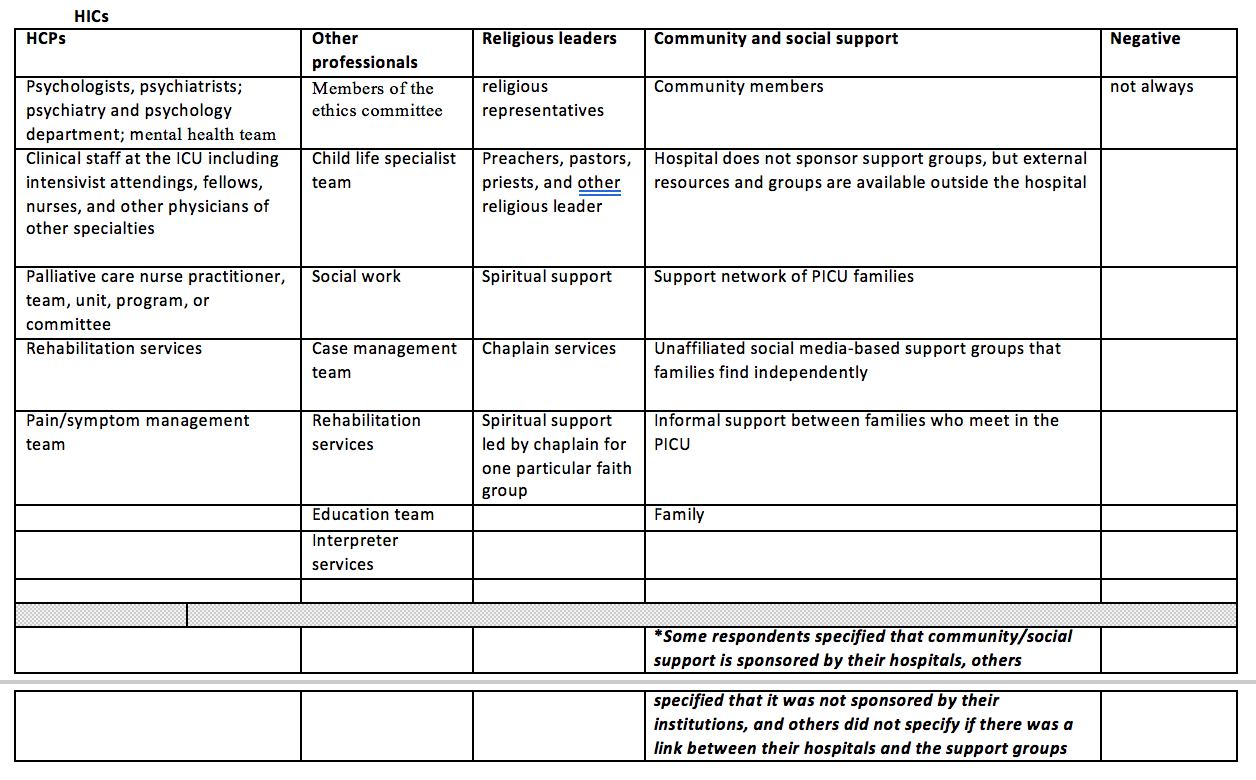
**

**
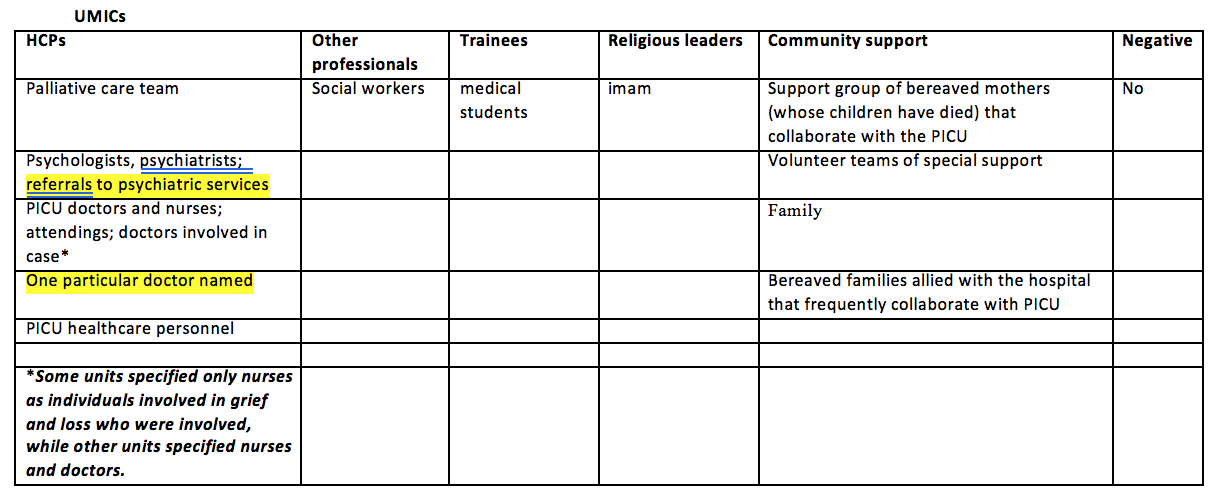
**

**
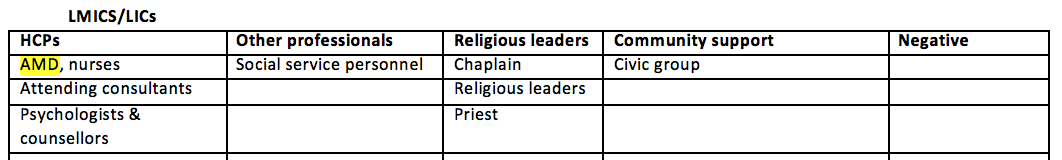
**

**Policies and guidelines established to ensure grief and loss support is provided to patients and families by country income level:**


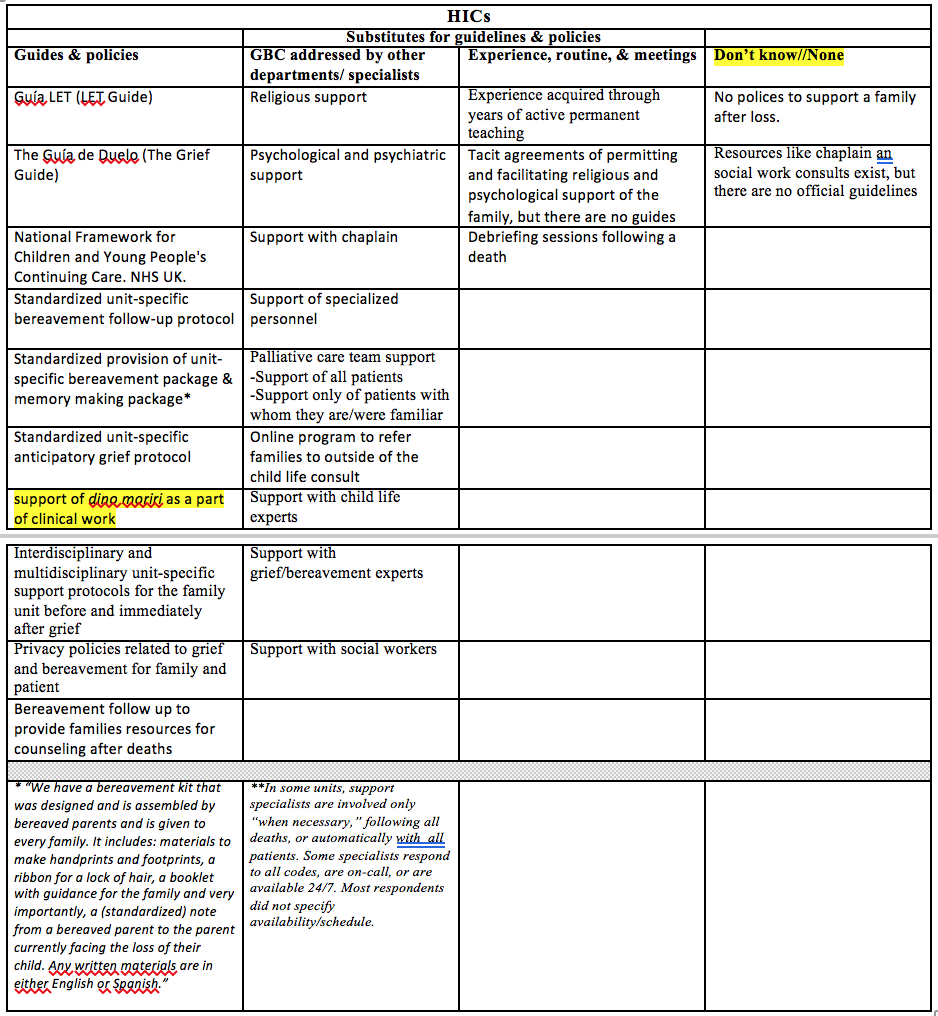


**
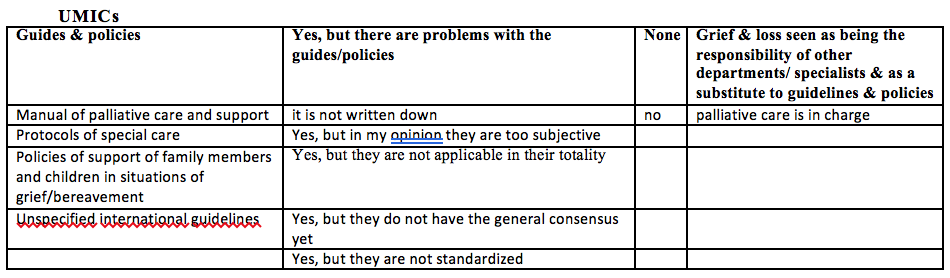
**

**LMICs/LICs units did not specify the polices/guidelines used.*
